# Dual Role of NRF2 in Pancreatic Precursor Lesions

**DOI:** 10.1158/2767-9764.CRC-25-0107

**Published:** 2025-06-11

**Authors:** Shu Ichimiya, Sung Shin Ahn, Maya S. Dixon, John P. O’Sullivan, Lela C. DeVine, Alex Chen, Takeo Yamamoto, Yoshinao Oda, Masafumi Nakamura, Iok In Christine Chio

**Affiliations:** 1 Department of Genetics and Development, Institute for Cancer Genetics, Columbia University Irving Medical Center, New York, New York.; 2 Herbert Irving Comprehensive Cancer Center, Columbia University Irving Medical Center, New York, New York.; 3 Department of Biomedical Engineering, Columbia University, New York, New York.; 4 Department of Biology, Barnard College, New York, New York.; 5 Department of Anatomic Pathology, Graduate School of Medical Sciences, Kyushu University, Fukuoka, Japan.; 6 Department of Surgery and Oncology, Graduate School of Medical Sciences, Kyushu University, Fukuoka, Japan.

## Abstract

Pancreatic ductal adenocarcinoma (PDA) arises from distinct precursor lesions, primarily pancreatic intraepithelial neoplasia (PanIN) and intraductal papillary mucinous neoplasm (IPMN). Unlike PanIN, IPMN is a cystic lesion detectable by imaging, providing an opportunity for early intervention. However, the molecular determinants guiding the formation of PanIN versus IPMN remain poorly understood. In this study, we uncover a previously unrecognized role for nuclear factor erythroid 2–related factor 2 (NRF2), a master regulator of redox homeostasis, in dictating pancreatic precursor lesion fate. Although NRF2 is known to promote PanIN formation and sustain PDA, we found that active NRF2 levels are significantly lower in human IPMN compared with PanIN and PDA. Using a conditional NRF2 knockout mouse model, we demonstrate that NRF2 loss significantly increases IPMN-like cystic tumor formation in *KRAS*^G12D^-mutant pancreatic epithelium, revealing an unexpected suppressive role of NRF2 in IPMN development. Mechanistically, NRF2 suppresses IPMN formation through redox-independent transcriptional repression of SAM pointed domain–containing Ets transcription factor and MUC6, key markers of IPMN. These findings establish NRF2 as a lesion-specific regulator of pancreatic tumorigenesis, providing new molecular insights into PDA progression and potential biomarkers for early detection and risk stratification.

**Significance:**

This study reveals a context-dependent role of NRF2 in pancreatic tumorigenesis, promoting PanIN progression while suppressing IPMN formation. These findings provide new insights into early lesion heterogeneity and highlight NRF2 status as a potential biomarker for risk stratification in pancreatic cancer.

## Introduction

Pancreatic ductal adenocarcinoma (PDA) is among the deadliest forms of cancer. Despite advances in surgical techniques and chemotherapy, the 5-year survival rate remains low (1). Because of its asymptomatic nature, PDA is often diagnosed at an advanced stage, with most cases deemed unresectable at the time of diagnosis (2). Although PDA can develop through different premalignant pathways, the most well studied is pancreatic intraepithelial neoplasia (PanIN). However, an alternative route to PDA involves cystic precursor lesions, including intraductal papillary mucinous neoplasms (IPMN; ref. 3). IPMN is characterized by a papillary proliferation of pancreatic ductal cells with mucin secretion, leading to ductal dilation (4). Unlike PanIN, IPMN is detectable via imaging, allowing for potential early intervention. However, the risk of malignancy varies among subtypes, with main-duct IPMN exhibiting a higher likelihood of progressing to invasive carcinoma compared with branch-duct IPMN (5, 6). Current surveillance criteria remain poorly defined, underscoring the need to better understand the molecular mechanisms that regulate IPMN formation and progression.

Several genetic mutations implicated in IPMN overlap with those observed in PDA (7). Among these, activating mutations in KRAS are highly prevalent in both PanIN and IPMN (8, 9). However, IPMN also harbors distinct genetic alterations, such as mutations in *GNAS* and *RNF43*, which are rare in PanIN and PDA (10). Notably, *GNAS* mutations are found in a significant proportion of IPMN cases and are particularly associated with the intestinal subtype (11). Genetically engineered mouse models have provided critical insights into the molecular pathways driving IPMN, demonstrating that combined *GNAS* and *KRAS* mutations induce cystic neoplasms that closely resemble human IPMN (12, 13). Although genetic alterations define the oncogenic landscape of IPMN, additional molecular regulators likely contribute to its development. In particular, redox homeostasis has emerged as a key player in pancreatic tumorigenesis (14, 15). nuclear factor erythroid 2–related factor 2 (NRF2), a master regulator of oxidative stress responses (16–18), is frequently upregulated in PDA, in which it promotes tumor growth and chemoresistance (14, 15).

Although NRF2 has been studied in PDA and PanIN, its role in IPMN remains unexplored. Given the distinct genetic landscape of IPMN and its potential for early detection, we sought to investigate whether NRF2 differentially regulates pancreatic precursor lesions. Using a pancreas-specific NRF2 knockout model (*Nfe2l2*^*flox/flox*^*;Pdx1-Cre*), we observed a significant increase in IPMN-like cystic tumor formation in *KRAS*^G12D^-mutant pancreatic epithelium, implicating NRF2 as a suppressor of IPMN. Unlike its pro-tumorigenic role in PanIN, NRF2 loss promoted IPMN formation through a redox-independent mechanism involving the transcriptional regulation of *Spdef* and *Muc6*, both key markers of IPMN. Furthermore, human tissue analysis revealed lower nuclear NRF2 expression in IPMN compared with PanIN and PDA, suggesting distinct regulatory mechanisms across pancreatic precursor lesions. By elucidating the context-dependent roles of NRF2 in pancreatic tumorigenesis, our findings provide new insights into the molecular determinants of IPMN formation. These results have potential implications for improving early detection strategies and developing targeted therapeutic approaches for PDA prevention.

## Materials and Methods

### Human patient samples

Pancreatic tissue was freshly collected from deidentified human patients undergoing resection of IPMN or PDA in compliance with IRB-AAR6773 and IRB-AAR7565. The study protocol was reviewed and approved by the Columbia University Institutional Review Board. The requirement for written informed consent was waived by the institutional review board. Patient samples include normal pancreas (*n* = 24), PanIN (*n* = 21), IPMN (*n* = 23), and PDA (*n* = 23).

### Animals

Conditional Nrf2^flox/flox^ mice were created on a C57BL/6 genetic background. LoxP sites were inserted into the introns before exon 4 and after exon 5 of the Nfe2l2 (Nrf2) allele. *KRAS*^*+/LSL-G12D*^, *Pdx1-Cre* (KC) strains in the C57Bl/6 background were interbred to obtain *Pdx1-Cre; KRAS*^*+/LSL-G12D*^*; Nrf2*^flox/flox^ (KCN) mice. All animal experiments were conducted in accordance with procedures approved by the Institutional Animal Care and Use Committee at Columbia University (AC-AABK554).

### Histologic evaluation of mouse pancreas

To evaluate the histologic characteristics of normal and neoplastic regions in KC and KCN mouse pancreata, hematoxylin and eosin slides of mice pancreata were comprehensively examined. The diagnosis of pancreatic neoplastic lesions in mice was based on the criteria established by Hruban and colleagues (19). The percentage areas of normal pancreatic tissue (normal), acinar-to-ductal metaplasia, PanIN (classified into PanIN-1a, PanIN-1b, PanIN-2, and PanIN-3), PDA, and IPMN were quantified. The area percentages for each of these regions were calculated relative to the total pancreatic area or the sum of neoplastic areas of each mouse. The analysis was conducted using QuPath software version 0.3.2 (20).

### IHC and immunofluorescence

Tissues were fixed in 10% neutral buffered formalin and embedded in paraffin. Sections were subjected to hematoxylin and eosin staining as well as IHC and immunofluorescent staining. Antigen retrieval was done in 10 mmol/L citrate buffer (pH 6). The following primary antibodies were used for IHC staining at the indicated dilution: MUC1 (Invitrogen, MA5-11202, RRID: AB_11000874, 1:200), MUC2 (Novus Biologicals, NB120-11197, RRID: AB_791261, 1:200), MUC2 (Proteintech, 27675-1-AP, RRID: AB_2880943, 1:2,000), NRF2 (Abcam, ab62352, RRID: AB_944418, 1:200), and NRF2 phospho S40 (Abcam, RRID: ab76026, AB_1524049, 1:400). The following primary antibodies were used for immunofluorescent staining at the indicated dilution: CK19 (DSHB, TROMA-III, RRID: AB_2133570, 1:100) and GNAS (Proteintech, 10150-2-AP, RRID: AB_2111668, 1:100). Quantitative analysis of NRF2 and pNRF2 expression intensity was performed using ImageJ2 software version 2.14.0/1.54f. (21).

### GNAS expression analysis

GNAS expression intensity analysis was performed using ImageJ software version 1.54f. Linear regions of interest of fixed length bisecting 101 arbitrarily chosen pancreatic ductal cells were assessed for GNAS fluorescent signal intensity as a function of distance for both KC and KCN IPMN tissue samples. Regions of interest were assigned across 41 composite images from six KCN mice and 34 composite images from five KC mice. Composite images were generated from hyperstacks of imaged tissue slices for each sample using the “Z project” function for maximal signal intensity.

### MRI analysis of pancreatic cysts

MRI scans were conducted using a Bruker BioSpec 9.4T Magnetic Resonance Imaging system (Bruker). Mice were anesthetized with a mixture of 1% to 2% isoflurane in medical air delivered through a nose cone. The isoflurane level was adjusted as needed to maintain a respiration rate between 40 and 70 breaths per minute, monitored with a respiratory sensor (SA Instruments). The body temperature was kept at approximately 37°C using a water-circulating heating pad. To start, low-resolution T1-weighted scout images were obtained to determine positioning. High-resolution T2-weighted images were then acquired using a rapid acquisition with relaxation enhancement sequence. Imaging parameters included repetition time = 4,873 milliseconds, echo time = 60 milliseconds, field of view = 34 × 34 mm, matrix size = 256 × 256, and slice thickness = 0.4 mm, with a total of 30 slices covering the abdominal region. The maximum intensity projection method was applied to enhance the visualization of cyst structures.

### Enzymatic dissociation of mouse pancreatic tissues

Harvested pancreata were rinsed with PBS, minced into 1 mm^3^ pieces on ice, and transferred to a 20 mL digestion buffer containing collagenase P (20 mg, Roche, cat. #11213857001), DNase I (200 µL 10 mg/mL, Roche, cat. #10104159001), FBS (400 µL), and Rho kinase inhibitor (Y-27632; 20 µL, Cayman Chemicals, cat. #10005583). The mixture was incubated for 75 minutes at 37°C with continuous rotation. Samples were then passed through a 70-μm cell strainer (CELLTREAT, cat. #229483), and the tube was rinsed with 20 mL of ice-cold PBS. Following centrifugation at 700 rpm for 5 minutes at 4°C, the pellet was resuspended in 1 mL of PBS containing 20 mmol/L HEPES, 2 mmol/L EDTA, 5 mmol/L MgCl_2_, 1% FBS, 50 μg/mL DNase I, and Rho kinase inhibitor (Y-27632). The cells were stained with DAPI, and viable cells were sorted using the BD Influx cell sorter. The viability of the cell samples was confirmed to be greater than 80%, and the cells were resuspended in PBS containing 10% FBS at a concentration of 1,000 cells/µL. The resulting single-cell suspension was used for single-cell RNA sequencing (scRNA-seq).

### scRNA-seq library preparation, sequencing, and analysis

Libraries for scRNA-seq were generated using the Chromium Next GEM Single Cell 3′ Reagent Kit v3.1 (10x Genomics). We aimed to profile 10,000 cells per library. All libraries were sequenced on the NovaSeq 6000 (Illumina) or DNBSEQ-G400 (MGI Tech). Raw sequencing reads were aligned to the mouse reference genome mm39 and processed into a matrix of unique molecular identifiers per cell barcode and gene using CellRanger v5.0.0 (10x Genomics). The resulting files were analyzed with the R v4.3.3 package “Seurat v5.0.3” (22). Low-quality cells (defined as those with nCount_RNA < 800, nFeature_RNA < 500, or percent.mt > 10) were excluded. Data from all samples were merged and normalized using the “SCTransform” (23) method for variance stabilization. Principal component analysis was performed with the “RunPCA” function, followed by clustering using the “FindNeighbors” and “FindClusters” functions. Clusters were visualized with dimensional reduction using the “UMAP” method.

### RNA sequencing data analysis

Existing RNA sequencing data from a study by Liffers and colleagues (24), utilizing RNA extracted from formalin-fixed, paraffin-embedded samples of human gastric IPMN and low-grade PanIN (LDG-PanIN) obtained from surgical specimens, were used (Gene Expression Omnibus series accession number GSE210351). Paired-end FASTQ files were uploaded to the RaNA-Seq platform (25), in which automatic differential expression analysis was performed to identify differentially expressed genes in gastric IPMN and LDG-PanIN.

### SDS-PAGE and immunoblot analysis

Protein lysates were prepared using 0.1% SDS lysis buffer in 50 mmol/L Tris pH 8, 0.5% deoxycholate, 150 mmol/L NaCl, 2 mmol/L EDTA, and 1% NP40, with one tablet of PhosSTOP (Roche, cat. #4906837001) and one tablet of cOmplete, Mini, EDTA-free protease inhibitor cocktail (Roche, cat. #11836170001) per 10 mL buffer and separated on 4% to 12% Bis-Tris SurePAGE gels (GenScript, cat. #M00653), then transferred onto a nitrocellulose membrane (Amersham Protran 0.45 μm, cat. #10600002), and incubated with the indicated antibodies for immunoblotting.

### Transcription factor motif analysis and genome annotation

The promoter sequences of the genes of interest were obtained using the UCSC Genome Browser. Transcription factor–binding motif data were downloaded from JASPAR, with MA0150.1 representing the *NFE2L2* (NRF2)-binding motif in *Homo sapiens* and MA0150.2 and MA0150.3 representing *Nfe2l2*-binding motifs in *Mus musculus*. Motif occurrences within the promoter regions were analyzed using the Find Individual Motif Occurrences tool. Predicted binding motifs were filtered based on a *P*-value threshold of <0.001. Motif enrichment scores were calculated as the sum of −log_10_ (*P* values) for predicted binding sites. To visualize the predicted binding sites, the start/end positions of identified motifs within the analyzed sequences were mapped and added as a custom track to the UCSC Genome Browser, enabling direct examination of NRF2-binding motifs in promoter regions.

### Statistical analysis

R software version 3.63, R package Gene Expression Signature (RRID: SCR_000455), R package ggpubr (RRID: SCR_021139), QuPath (RRID: SCR_018257), and GraphPad Software version 9.0 (RRID: SCR_002798) were used to assess the data. Data are presented as the mean ± SD. A Student *t* test was used to compare the differences between two groups.

### Data availability

All data supporting the findings of this study are available in the article and its Supplementary Material and are available upon request from the corresponding author. scRNA-seq data have been deposited into the Gene Expression Omnibus repository under accession number GSE289513.

## Results

### Distinct patterns of NRF2 expression in human pancreatic lesions

**Figure 1:**
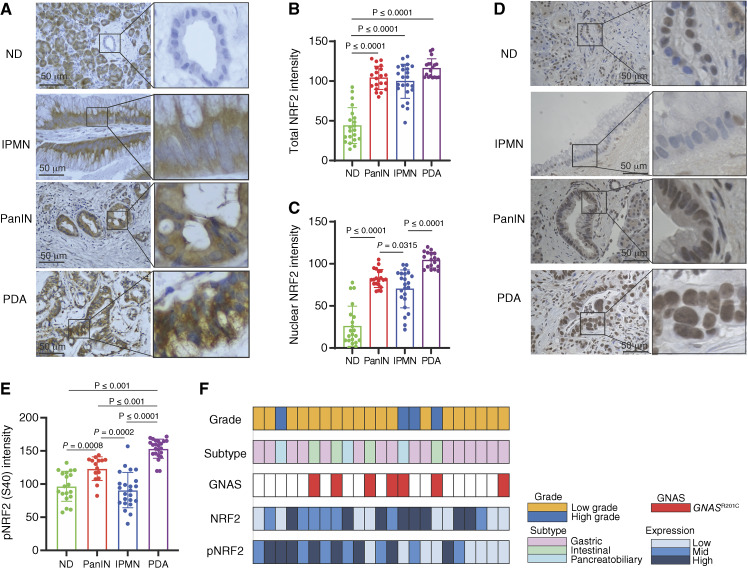
Distinct patterns of NRF2 expression in human pancreatic lesions. **A,** Representative image showing IHC staining for NRF2 in normal human pancreatic ducts, PanIN, IPMN, and PDA. **B,** Quantitative analysis of total NRF2 intensity in ND (*n* = 21), PanIN (*n* = 20), IPMN (*n* = 23), and PDA (*n* = 18). A one-way ANOVA comparing all groups with *F* = 62.74 and *P* < 0.0001. **C,** Quantitative analysis of nuclear NRF2 intensity in ND (*n* = 21), PanIN (*n* = 20), IPMN (*n* = 23), and PDA (*n* = 18). A one-way ANOVA comparing all groups with *F* = 66.69 and *P* < 0.0001. **D,** Representative IHC images of phosphorylated NRF2 (pNRF2^Ser40^) in human ND, PanIN, IPMN, and PDA. **E,** Quantitative analysis of pNRF2^Ser40^ staining intensity in human ND (*n* = 19), PanIN (*n* = 15), IPMN (*n* = 23), and PDA (*n* = 23). A one-way ANOVA comparing all groups with *F* = 40.73 and *P* < 0.0001. **F,** Histologic grade, subtype, *GNAS*^R201C^ mutation status, NRF2 nuclear staining intensity, and pNRF2 nuclear staining intensity in all human IPMN specimens analyzed. Error bars in this figure represent means ± SDs. A Student *t* test was conducted. Unless noted otherwise, no significant differences were found between the groups. ND, normal human pancreatic ducts.

We conducted IHC analysis on a panel of patient-derived specimens, including normal pancreas (*n* = 24), PanIN (*n* = 21), IPMN (*n* = 23), and PDA (*n* = 23; Table 1). Consistent with prior studies (18–22), total NRF2 expression was elevated in all preneoplastic and neoplastic lesions compared with normal ductal cells (Fig. 1A and B). Nuclear NRF2 expression, a marker of active NRF2 (17), was highest in PDA, significantly exceeding levels observed in preneoplastic lesions. Notably, NRF2 expression in IPMN lesions was predominantly cytosolic, similar to non-transformed acinar cells, in contrast to the nuclear localization observed in PanIN and PDA (Fig. 1A and C).

**Table 1:** Patient samples used for NRF2 immunostaining

Normal			PanIN			IPMN			PDA		
Sample ID	Age	Sex	Sample ID	Age	Sex	Sample ID	Age	Sex	Sample ID	Age	Sex
cchN10	55	F	cchN12	61	M	cchP8	64	F	cchT2	88	M
cchN11B	59	M	cchN14	66	F	cchP11	54	M	cchT7	75	F
cchN12	61	M	cchN15	69	M	cchP12	72	M	cchT13	85	F
cchN14	66	F	cchN17	67	F	cchP13	73	F	cchT14	71	M
cchN15	69	M	cchN22	69	F	cchP14	80	F	cchT17	68	M
cchN17	67	F	cchN28	64	M	cchP15	67	F	cchT19	68	M
cchN21	75	M	cchN36	74	F	cchP16	82	F	cchT22	61	M
cchN22	69	F	cchN41	75	F	cchP20	64	M	cchT26	69	M
cchN27	72	F	cchN46	73	F	cchP22	79	F	cchT32	75	M
cchN30	80	M	cchN51	79	F	cchP23	75	M	cchT35	84	M
cchN31	69	F	cchN52	63	M	cchP25	67	M	cchT36	84	F
cchN32	39	M	cchN104	72	M	cchP26	75	F	cchT40	64	M
cchN34	84	F	cchN110	82	F	cchP27	69	M	cchT46	68	M
cchN35	74	M	cchN115	64	M	cchP27 (PB)	69	M	cchT49	80	M
cchN36	74	F	cchN125	67	M	cchP32	79	M	cchT51	39	M
cchN37	40	F	cchN129	69	M	cchP33	72	M	cchT56	77	F
cchN38	63	M	cchN138	72	M	cchP39	72	F	cchT57	77	F
cchN41	75	F	cchN166	65	F	cchP44	79	F	cchT58	69	M
cchN42	55	F	cchP8	64	F	cchP46	71	F	cchT59	84	F
cchN43	75	F	P20	64	M	cchP49	79	F	cchT60	74	M
cchN46	73	F	cchP34	62	F	cchP50	73	F	cchT66	55	F
cchN47	67	M				cchP54	80	M	cchT74	49	F
cchN49	49	F				cchP55	72	M	cchT75	79	F
cchN52	63	M									

List of samples derived from patients that were used to determine NRF2 expression. Samples were classified as normal human pancreatic ducts (*n* = 24), PanIN (*n* = 21), IPMN (*n* = 23), and PDA (*n* = 23). Sample cchP27 (PB) refers to a pancreatobiliary sample.

The phosphorylation of NRF2 at Ser40, a posttranslational modification involved in antioxidant response element (ARE)–mediated transcription (26, 27), was upregulated in PanIN and PDA compared with normal ducts but remained unchanged in IPMN (Fig. 1D and E). The expression of total, nuclear, and phosphorylated NRF2 did not correlate with the histologic grade of the IPMN lesions (Fig. 1F; Supplementary Fig. S1A–S1C) or the mutational status of *GNAS* (R201C), the most frequently observed genetic alteration in IPMN (Fig. 1F; Supplementary Fig. S1D–S1F; ref. 28). However, higher-grade lesions exhibited a trend toward increased nuclear (Supplementary Fig. S1A) and active (Supplementary Fig. S1C) NRF2 expression. Additionally, NRF2 expression did not correlate with the histologic subtypes of IPMN (gastric, intestinal, and pancreatobiliary) as defined by MUC1 and MUC2 expression patterns (Fig. 1F; Supplementary Fig. S1G). These findings suggest that NRF2 may have distinct functional roles in PanIN and IPMN, influencing their divergent progression pathways.

### Deletion of NRF2 in mouse pancreatic epithelium promotes IPMN-like lesions

**Figure 2:**
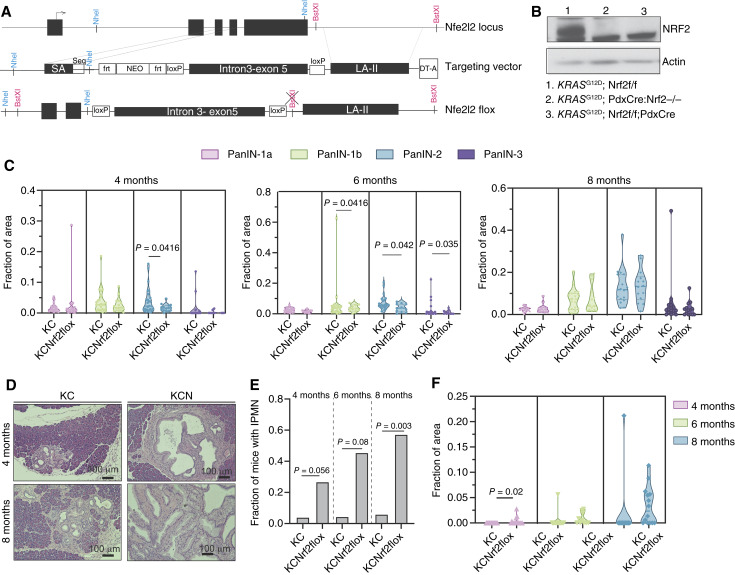
Deletion of NRF2 in mouse pancreatic epithelium promotes IPMN-like lesions. **A,** Targeting strategy of conditional NRF2 deletion strain. **B,** Immunoblot analysis of NRF2 expression in pancreatic ductal organoid lysates. **C,** Violin plots illustrating the area of neoplastic lesions in age-matched cohorts (4, 6, and 8 months) for KC and KCN pancreata. The cohorts comprised 25 KC and 15 KCN mice at 4 months, 23 KC and 11 KCN mice at 6 months, and 17 KC and 14 KCNrf2flox mice at 8 months of age. **D,** Representative images of hematoxylin and eosin staining of KC and KCN pancreata at 4 and 8 months of age. **E,** Statistical analysis (Fisher exact test) of the incidence of IPMN-like cystic tumors in KC and KCN mice was conducted. The cohorts included 25 KC and 15 KCN mice at 4 months, 23 KC and 11 KCNrf2flox mice at 6 months, and 17 KC and 14 KCNrf2flox mice at 8 months of age. **F,** Violin plots illustrating the area of IPMN lesions in age-matched cohorts (4, 6, and 8 months) for KC and KCN pancreata. The cohorts comprised 25 KC and 15 KCN mice at 4 months, 23 KC and 11 KCN mice at 6 months, and 17 KC and 14 KCNrf2flox mice at 8 months of age. The error bars in this figure represent means ± SDs. A Student *t* test was conducted unless stated otherwise. If not specified, no significant differences were observed between the two groups.

To investigate the role of NRF2 in pancreatic preneoplasia, we generated a conditional *Nfe2l2* knockout strain (*Nrf2*flox) to enable targeted deletion of NRF2 in the pancreatic epithelium. These mice were crossed with KC (*KRAS*^G12D^;*Pdx1*-Cre) mutants to generate KCNrf2flox compound mutants (KCN; Fig. 2A), in which NRF2 expression was ablated specifically in pancreatic epithelial cells (Fig. 2B).

We analyzed the incidence of neoplastic lesions in KC and KCN mice at 4, 6, and 8 months of age. As previously observed in full-body *Nfe2l2* knockout models (15), conditional deletion of NRF2 in the pancreatic epithelium also led to a significant reduction in PanIN formation in KCN mice at 4 and 6 months (Fig. 2C; Supplementary Fig. S2A and S2B). In contrast, KCN mice exhibited a markedly higher frequency of IPMN-like cystic tumors compared with KC mice (Fig. 2D and E), and the proportion of mice with these lesions increased with age. Although the neoplastic area occupied by IPMN lesions was greater in KCN mice at 4 months, the lesion size among affected mice was comparable between genotypes in later time points (Fig. 2F; Supplementary Fig. S2B). These findings demonstrate that NRF2 deletion in *KRAS*^G12D^-mutant pancreatic epithelium not only suppresses PanIN formation, as seen in full-body knockout models, but also increases the likelihood of developing IPMN-like cystic lesions. This phenotypic divergence highlights the dual role of NRF2 in pancreatic preneoplastic lesion development and suggests its potential influence on lineage-specific tumorigenic trajectories.

### NRF2 suppresses IPMN formation through a redox-independent mechanism

As a central regulator of intracellular redox homeostasis (18), NRF2 mitigates oxidative stress, raising the question of whether reactive oxygen species (ROS) influence the distinct progression of *KRAS*^G12D^-mutant pancreatic epithelial cells into PanIN or IPMN lesions. To investigate this, we examined whether NRF2 deletion promotes IPMN formation by altering global ROS levels, following established protocols to perturb intrapancreatic ROS (15). KC and KCN mice were randomized into three groups receiving water, buthionine sulfoximine (BSO), or N-acetylcysteine (NAC) for 6 months. BSO inhibits γ-glutamylcysteine synthetase, depleting glutathione and consequently increasing intracellular ROS (29, 30). Conversely, NAC, a glutathione precursor, reduces ROS levels (31).

**Figure 3:**
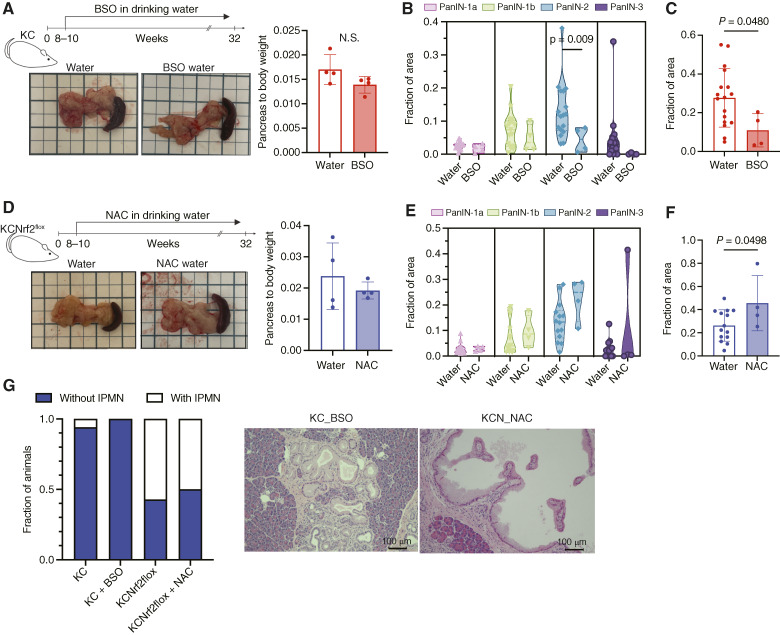
NRF2 suppresses IPMN formation in a redox-independent manner. **A,** Experimental timeline and representative images of the pancreas and spleen collected from KC mice treated with either water or BSO (20 mmol/L in drinking water). Quantification of the pancreas relative to the body weight of these animals. **B,** Violin plots illustrating the area of neoplastic lesions in the pancreata from KC mice treated with BSO (*n* = 4) or water (*n* = 17). **C,** Statistical analysis of total PanIN area in KC mice treated with BSO (*n* = 4) or water (*n* = 17). **D,** Experimental timeline and representative images of the pancreas and spleen collected from KCN mice treated with either water or NAC (30 mmol/L in drinking water). Quantification of the pancreas relative to the body weight of these animals. **E,** Violin plots illustrating the area of neoplastic lesions in the pancreata from KCN mice treated with NAC (*n* = 4) or water (*n* = 14). **F,** Statistical analysis of total PanIN area in KCN mice treated with water (*n* = 14) or NAC (*n* = 4). **G,** Incidence of IPMN-like cystic tumors in mice treated with NAC (*n* = 4), BSO (*n* = 4), or water (KC, *n* = 17 and KCN, *n* = 14). Representative hematoxylin and eosin images of pancreata from KC mice administered BSO and KCN mice administered NAC. Error bars in this figure represent means ± SDs. A Student *t* test was conducted. Unless noted otherwise, no significant differences were found between the groups.

Systemic BSO administration did not significantly affect pancreas-to-body-weight ratios (Fig. 3A), but it decreased PanIN formation in KC mice (Fig. 3B and C), indicating that elevated ROS levels hinder PanIN development in *KRAS*^G12D^-mutant pancreatic epithelial cells. Conversely, NAC administration did not affect pancreas-to-body-weight ratios (Fig. 3D) but significantly enhanced PanIN formation in KCN mice (Fig. 3E and F), supporting a role for NRF2 in promoting PanIN formation by mitigating oxidative stress. However, neither BSO nor NAC treatment altered the incidence of IPMN-like lesions in either KC or KCN animals (Fig. 3G). These results suggest that although NRF2 facilitates PanIN formation via its redox-regulating functions, consistent with published findings (15), its suppression of IPMN development occurs through a distinct, redox-independent mechanism.

### NRF2 loss enhances IPMN-associated gene signatures and SAM pointed domain–containing Ets transcription factor pathways

**Figure 4:**
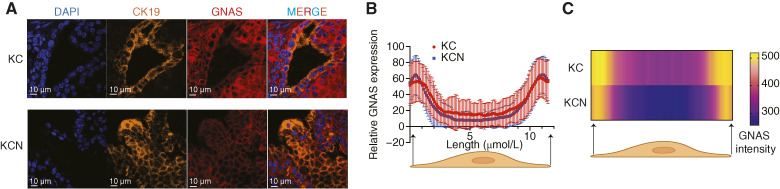
NRF2 loss does not alter GNAS intracellular localization. **A,** Representative images of immunofluorescence staining of KC and KCN pancreata. **B,** Quantification of GNAS staining intensity across the width of a pancreatic ductal cell. Using ImageJ, 101 random pancreatic ductal cells were surveyed and analyzed. **C,** Distribution of GNAS expression across the width of a pancreatic ductal cell. Using ImageJ, 101 random pancreatic ductal cells were surveyed and analyzed. Error bars in this figure represent mean ± SD. A Student *t* test was conducted. Unless noted otherwise, no significant differences were found between the groups.

To identify the redox-independent mechanisms by which NRF2 regulates IPMN formation, we examined *GNAS* (R201C), the most common genetic alteration in IPMN (28). GNAS encodes a critical mediator of G protein–coupled receptor signaling, initiating downstream adenylyl cyclase activation (32). As the cytoplasmic diffusion of GNAS has been linked to its signaling activity (33), we assessed GNAS localization in pancreatic tissues via immunofluorescence staining. We observed no significant differences in GNAS intracellular distribution between KC and KCN mice (Fig. 4). However, NRF2 deletion led to a modest increase in GNAS membrane localization (Fig. 4B and C). Given that membrane localization of GNAS is typically associated with an inactive state, these observations indicate that the formation of cystic neoplasms in NRF2-deficient pancreata likely occurs through mechanisms independent of canonical GNAS activation.

**Figure 5:**
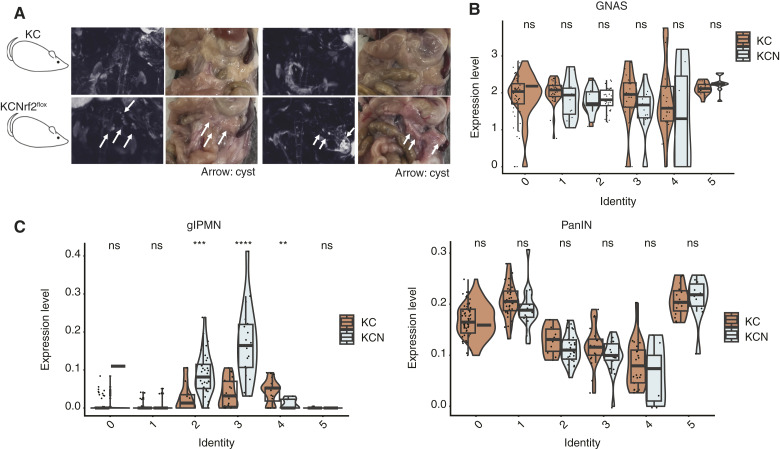
NRF2 loss promotes IPMN-like gene signatures. **A,** Magnetic resonance and brightfield images of KC and KCN pancreata (*n* = 2 each) harvested for scRNA-seq. **B,** Violin plot of *GNAS* expression across ductal cell clusters. **C,** Violin plot of gastric IPMN and PanIN signature expression across ductal cell clusters. Error bars in this figure represent means ± SDs. A Student *t* test was conducted. **, *P* = 0.00356; ***, *P* = 0.00098; ****, *P* < 0.0001; ns, not significant. Unless otherwise noted, no significant differences were observed between the two groups.

To further characterize the molecular impact of NRF2 loss, we conducted scRNA-seq on the pancreata of 8-month-old mice with MRI-confirmed cystic lesions (Fig. 5A; Supplementary Fig. S3A). We noted a significant dropout of epithelial cells, likely attributed to a loss of epithelial cell viability during tissue dissociation. Of the epithelial cells captured in this analysis, pancreatic ductal cells were identified using *Krt19* and *Cpa1* markers (Supplementary Fig. S3B and S3C) and categorized into six distinct subclusters (Supplementary Fig. S3D). Notably, clusters 2 and 3 were predominantly enriched in KCN pancreata (Supplementary Fig. S3D). To assess the relevance of these clusters to human disease, we applied publicly available datasets of human IPMN and LDG-PanIN (GSE210351; ref. 24) to identify differentially expressed genes specific to IPMN and LDG-PanIN (Supplementary Fig. S3E). Mapping these gene sets onto the murine ductal cell clusters by UCell (34) revealed that the IPMN signature was primarily associated with clusters 2 and 3 (Supplementary Fig. S3F). Although *GNAS* expression remained unchanged between KC and KCN pancreata (Fig. 5B), IPMN-associated gene sets were significantly upregulated in KCN pancreata compared with KC (Fig. 5C). These findings reinforce the relevance of NRF2 loss to human IPMN pathophysiology and suggest that NRF2 modulates distinct molecular pathways that drive lineage-specific pancreatic tumorigenesis independently of *GNAS* activation.

**Figure 6:**
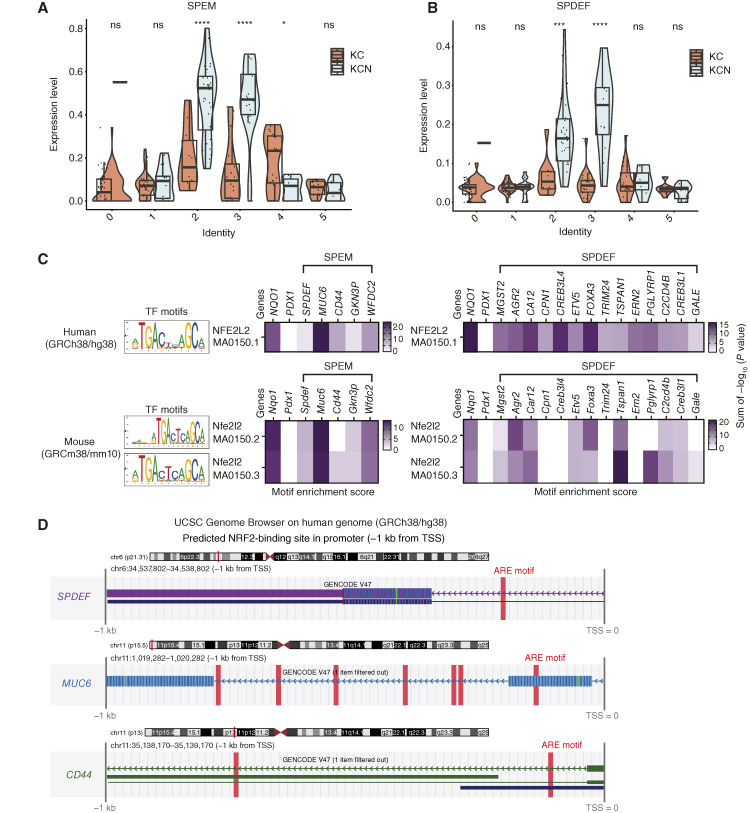
NRF2 loss promotes transcriptional activation of SPEM and SPDEF gene signatures. **A,** Violin plot of SPEM signature expression across ductal cell clusters. Error bars in this figure represent means ± SDs. A Student *t* test was conducted. ****, *P* < 0.0001; *, *P* = 0.017; ns, not significant. Unless otherwise noted, no significant differences were observed between the two groups. **B,** Violin plot of SPEDF signature expression across ductal cell clusters. Error bars in this figure represent means ± SDs. A Student *t* test was conducted. ***, *P* = 0.00013; ****, *P* < 0.0001; ns, not significant. Unless otherwise noted, no significant differences were observed between the two groups. **C,** Transcription factor motif analysis for NRF2 (NFE2L2) binding (MA0150.1) in human *NQO1*, *PDX1*, SPEM and SPDEF gene signatures (top) and for *Nfe2l2* (MA0150.2 and MA0150.3) in mouse *Nqo1*, *Pdx1*, SPEM and SPDEF signatures (bottom) using Find Individual Motif Occurrences. Motif enrichment scores represent the sum of −log_10_ (*P* values) for predicted binding sites. **D,** UCSC Genome Browser tracks displaying predicted ARE motifs in the promoters of SPDEF, MUC6, and CD44 [−1 kb from their transcription start sites (TSS)]. Predicted binding sequences, locations, and *P* values for ARE motifs in the promoters of genes analyzed in **C** and **D** are provided in Table 2.

A recent study identified markers of gastric spasmolytic polypeptide–expressing metaplasia (SPEM) in human IPMN, with SAM pointed domain–containing Ets transcription factor (SPDEF), CREB3L1, and CREB3L4 emerging as key transcriptional regulators of this phenotype (bioRxiv 2024.02.25.581948). Consistent with these findings, clusters 2 and 3—enriched in KCN pancreatic ductal cells—exhibited significant enrichment for both SPEM (Fig. 6A) and SPDEF (Fig. 6B) gene signatures. To determine whether NRF2 directly regulates this pathway, we performed transcription factor motif analysis on the promoters of SPEM and SPDEF target genes in both human and mouse genomes. As expected, the canonical ARE, the NRF2-binding motif, was significantly enriched within the SPEM and SPDEF gene sets at levels comparable with those observed in *Nqo1*, a well-established NRF2 target gene (Fig. 6C; Table 2; ref. 18). Notably, within 1 kb upstream of their transcription start sites, key markers of pyloric metaplasia, including *Muc6*, *Spdef*, and *Cd44*, contained ARE motifs (Fig. 6D; Table 2).

**Table 2:** Predicted NRF2-binding motifs identified using Find Individual Motif Occurrences analysis

h MA0150.1									
Motif ID	Alt ID	Sequence name	Strand	Start	End	*P* value	−Log_10_ (*P* value)	*q* value	Matched sequence
MA0150.1	NFE2L2	AGR2	+	−87	−77	0.00012	3.920818754	0.692	ctgagttagca
MA0150.1	NFE2L2	AGR2	+	−21	−11	0.000552	3.258060922	0.692	gagactcagct
MA0150.1	NFE2L2	AGR2	+	−624	−614	0.000803	3.095284455	0.715	atggcaaggca
MA0150.1	NFE2L2	C2CD4B	−	−789	−779	0.000237	3.625251654	0.692	GTTAGAAAGCA
MA0150.1	NFE2L2	C2CD4B	+	−45	−35	0.000376	3.424812155	0.692	gggactcagcc
MA0150.1	NFE2L2	CA12	+	−949	−939	0.000575	3.240332155	0.692	atgccactgca
MA0150.1	NFE2L2	CA12	+	−357	−347	0.000667	3.175874166	0.692	ctgtctaagca
MA0150.1	NFE2L2	CA12	+	−309	−299	0.000676	3.170053304	0.692	gtgacagacca
MA0150.1	NFE2L2	CA12	−	−201	−191	0.000971	3.01278077	0.724	CTGACCCAGGA
MA0150.1	NFE2L2	CD44	−	−115	−105	1.16E-06	5.935542011	0.0237	GTGACTAAGCA
MA0150.1	NFE2L2	CD44	+	−746	−736	0.000485	3.314258261	0.692	ATGAATGAGCA
MA0150.1	NFE2L2	CPN1	−	−742	−732	0.000425	3.37161107	0.692	ATGATCATGCC
MA0150.1	NFE2L2	CPN1	+	−467	−457	0.000635	3.197226275	0.692	atgacactgaa
MA0150.1	NFE2L2	CREB3L1	−	−660	−650	0.000248	3.605548319	0.692	GTGATCCGGCC
MA0150.1	NFE2L2	CREB3L1	−	−285	−275	0.0008	3.096910013	0.715	ATGAGGGAGCT
MA0150.1	NFE2L2	CREB3L4	−	−934	−924	0.000292	3.534617149	0.692	ATGAGGAGGCA
MA0150.1	NFE2L2	CREB3L4	−	−546	−536	0.000421	3.375717904	0.692	GTGACTCGGAA
MA0150.1	NFE2L2	CREB3L4	+	−723	−713	0.000465	3.332547047	0.692	gtgaatgagca
MA0150.1	NFE2L2	CREB3L4	+	−79	−69	0.000579	3.237321436	0.692	aggactcagct
MA0150.1	NFE2L2	ERN2	+	−238	−228	0.000822	3.085128182	0.715	atgaaccggca
MA0150.1	NFE2L2	ERN2	−	−659	−649	0.000939	3.027334408	0.724	GTGCCATTGCA
MA0150.1	NFE2L2	ERN2	+	−780	−770	0.000939	3.027334408	0.724	gtgccattgca
MA0150.1	NFE2L2	ETV5	+	−362	−352	5.72E-05	4.242603971	0.692	ctgactcagct
MA0150.1	NFE2L2	ETV5	+	−966	−956	0.000198	3.70333481	0.692	ctgagtctgca
MA0150.1	NFE2L2	FOXA3	−	−936	−926	0.000157	3.804100348	0.692	GGGACTCAGCA
MA0150.1	NFE2L2	FOXA3	+	−277	−267	0.000222	3.653647026	0.692	atgatgaagcc
MA0150.1	NFE2L2	FOXA3	−	−188	−178	0.000513	3.289882635	0.692	GTGATTCAGGA
MA0150.1	NFE2L2	FOXA3	−	−199	−189	0.000766	3.11577123	0.715	GTGAGGGAGCT
MA0150.1	NFE2L2	GALE	−	−952	−942	0.000268	3.571865206	0.692	CTGAGTAAGCC
MA0150.1	NFE2L2	GKN3P	−	−113	−103	0.000258	3.588380294	0.692	ATGATAAGGCG
MA0150.1	NFE2L2	MGST2	+	−427	−417	0.000676	3.170053304	0.692	gtgacagatca
MA0150.1	NFE2L2	MGST2	+	−457	−447	0.000814	3.089375595	0.715	atgatcgtgcc
MA0150.1	NFE2L2	MGST2	−	−895	−885	0.000918	3.037157319	0.724	GTAACATGGCA
MA0150.1	NFE2L2	MUC6	+	−142	−132	0.000115	3.93930216	0.692	CTGATAAAGCA
MA0150.1	NFE2L2	MUC6	−	−404	−394	0.000138	3.860120914	0.692	CTGACCCAGCT
MA0150.1	NFE2L2	MUC6	+	−293	−283	0.000246	3.609064893	0.692	TTGACCCAGCA
MA0150.1	NFE2L2	MUC6	+	−781	−771	0.000336	3.473660723	0.692	ATGCCCCAGCA
MA0150.1	NFE2L2	MUC6	−	−660	−650	0.000421	3.375717904	0.692	GTGTCTCTGCA
MA0150.1	NFE2L2	MUC6	+	−307	−297	0.00047	3.327902142	0.692	CTGACAGTGCC
MA0150.1	NFE2L2	MUC6	−	−544	−534	0.000838	3.076755981	0.715	ATCACCCAGCG
MA0150.1	NFE2L2	NQO1	+	−456	−446	2.05E-07	6.688246139	0.00839	GTGACTCAGCA
MA0150.1	NFE2L2	NQO1	−	−120	−110	0.00054	3.26760624	0.692	CTGAGGCTGCA
MA0150.1	NFE2L2	NQO1	−	−957	−947	0.00055	3.259637311	0.692	GTGCCACTGCA
MA0150.1	NFE2L2	NQO1	−	−593	−583	0.000575	3.240332155	0.692	ATGCCACTGCA
MA0150.1	NFE2L2	PGLYRP1	+	−532	−522	0.000376	3.424812155	0.692	gtgtctcagcc
MA0150.1	NFE2L2	PGLYRP1	+	−172	−162	0.000826	3.083019953	0.715	gtgacccagac
MA0150.1	NFE2L2	PGLYRP1	−	−103	−93	0.00089	3.050609993	0.724	CTGGCCCAGCA
MA0150.1	NFE2L2	SPDEF	+	−208	−198	0.000631	3.199970641	0.692	GTGGCTATGCA
MA0150.1	NFE2L2	TRIM24	−	−785	−775	0.0008	3.096910013	0.715	ATCATTAAGCA
MA0150.1	NFE2L2	TRIM24	−	−263	−253	0.000971	3.01278077	0.724	CTGATTGGGCC
MA0150.1	NFE2L2	TSPAN1	−	−729	−719	0.000639	3.194499142	0.692	CTGACACACCA
MA0150.1	NFE2L2	TSPAN1	−	−859	−849	0.000855	3.068033885	0.715	ATGACACAGTT
MA0150.1	NFE2L2	WFDC2	−	−426	−416	0.000264	3.578396073	0.692	ATGAGAGGGCA
MA0150.1	NFE2L2	WFDC2	−	−629	−619	0.000361	3.442492798	0.692	CTGACTGTGCC
MA0150.1	NFE2L2	WFDC2	−	−712	−702	0.000366	3.436518915	0.692	ATGACAAAGGA

m MA0150.2							
Sequence name	Strand	Start	End	*P* value	−Log_10_ (*P* value)	*q* value	Matched sequence
Agr2	+	−90	−76	0.000233	3.632644	0.881	gcttctgagttagca
Agr2	−	−465	−451	0.000776	3.110138	1	TACAGTTATACAGCT
Agr2	+	−485	−471	0.000822	3.085128	1	tcttgtgatcgagca
Agr2	+	−439	−425	0.00087	3.060481	1	aactgtgaatcatcc
Agr2	+	−931	−917	9.92E−04	3.003488	1	tataatgatataaca
C2cd4b	+	−371	−357	0.000449	3.347754	0.957	gcttatgagtcaggt
C2cd4b	−	−739	−725	0.000642	3.192465	1	ATGAATGACTTGGGG
Car12	+	−240	−226	0.0003	3.522879	0.881	ggcagtgacagacca
Car12	+	−506	−492	0.000471	3.326979	0.957	tcctgtgaatttgca
Car12	−	−344	−330	0.000879	3.056011	1	GTGAATTACACATCA
Cd44	−	−158	−144	3.51E−04	3.454693	0.881	GAGAATGACTGAGTT
Creb3l1	+	−183	−169	0.000747	3.126679	1	agagctgagccagcc
Creb3l1	−	−306	−292	0.000943	3.025488	1	AGGACTGACTAGGAA
Etv5	+	−252	−238	4.80E−05	4.318759	0.445	ttcactgactcagct
Etv5	−	−435	−421	6.84E−04	3.164944	1	CTCGCTGACCCTGCC
Foxa3	+	−885	−871	3.10E−05	4.508638	0.428	cgccgtgacacggcg
Foxa3	+	−512	−498	0.000291	3.536107	0.881	gaccctgactctgta
Foxa3	−	−374	−360	0.000661	3.179799	1	GAGGTTGACAGAGCT
Foxa3	−	−620	−606	0.000729	3.137272	1	CAACGTTAGCCAGCC
Gkn3p	−	−158	−144	0.000351	3.454693	0.881	GAGAATGACTGAGTT
Mgst2	−	−55	−41	8.03E−04	3.095284	1	AGAGTTGAGTCAACA
Muc6	+	−402	−388	0.000191	3.718967	0.881	cacagtgatctggca
Muc6	+	−497	−483	0.000286	3.543634	0.881	ctggattacctagca
Muc6	+	−237	−223	0.000288	3.540608	0.881	agcactgataaagca
Muc6	−	−741	−727	0.000315	3.501689	0.881	TGGGCTGAGTGAGCT
Nqo1	+	−440	−426	1.36E−06	5.866461	0.0562	cacagtgagtcggca
Nqo1	−	−448	−434	0.000369	3.432974	0.881	CACTGTGACTCTAGA
Nqo1	+	−364	−350	0.000607	3.216811	1	acggctgagtgagga
Pglyrp1	−	−388	−374	3.17E−04	3.498941	0.881	TAGGATGACTTTGAA
Pglyrp1	+	−178	−164	0.000383	3.416801	0.881	cctgatgagagagcc
Pglyrp1	−	−652	−638	0.000496	3.304518	0.957	GTGTCTGAATCAGCC
Pglyrp1	−	−134	−120	9.47E−04	3.02365	1	GGCCGTGATGGAGCC
Spdef	+	−696	−682	1.93E−05	4.714443	0.399	ccccgtgattcagct
Tspan1	+	−360	−346	0.000509	3.293282	0.957	ccccgtgactgagtt
Tspan1	+	−217	−203	0.000718	3.143876	1	gctcctgacactgct
Tspan1	+	−644	−630	0.000762	3.118045	1	ggttgtgagccacca
Tspan1	+	−453	−439	0.000762	3.118045	1	ggttgtgagccacca
Wfdc2	−	−658	−644	6.06E−05	4.217527	0.445	GGTGGTGACTATGCA
Wfdc2	−	−765	−751	6.45E−05	4.19044	0.445	CACCATGACTAAGGC

m MA0150.3									
Motif ID	Alt ID	Sequence name	Strand	Start	End	*P* value	−Log_10_ (*P* value)	*q* value	Matched sequence
MA0150.3	Nfe2l2	Agr2	+	−86	−76	6.34E−05	4.19791074	0.527	ctgagttagca
MA0150.3	Nfe2l2	Agr2	+	−481	−471	0.000271	3.56703071	0.885	gtgatcgagca
MA0150.3	Nfe2l2	Agr2	+	−927	−917	0.000889	3.05109824	0.919	atgatataaca
MA0150.3	Nfe2l2	C2cd4b	+	−367	−357	0.000143	3.84466396	0.851	atgagtcaggt
MA0150.3	Nfe2l2	C2cd4b	−	−739	−729	0.000605	3.21824463	0.919	ATGACTTGGGG
MA0150.3	Nfe2l2	C2cd4b	+	−608	−598	0.000859	3.06600684	0.919	ctgacccggga
MA0150.3	Nfe2l2	Car12	+	−502	−492	0.000266	3.57511836	0.885	gtgaatttgca
MA0150.3	Nfe2l2	Car12	+	−236	−226	0.000343	3.46470588	0.885	gtgacagacca
MA0150.3	Nfe2l2	Car12	−	−344	−334	0.000746	3.12726117	0.919	ATTACACATCA
MA0150.3	Nfe2l2	Car12	+	−189	−179	0.000988	3.00524306	0.956	ctgactttcca
MA0150.3	Nfe2l2	Cd44	−	−158	−148	0.000624	3.20481541	0.919	ATGACTGAGTT
MA0150.3	Nfe2l2	Creb3l1	+	−965	−955	0.000525	3.2798407	0.919	ctgagtctgga
MA0150.3	Nfe2l2	Creb3l1	+	−179	−169	0.000597	3.22402567	0.919	ctgagccagcc
MA0150.3	Nfe2l2	Etv5	+	−248	−238	2.84E−05	4.54668166	0.398	ctgactcagct
MA0150.3	Nfe2l2	Etv5	−	−435	−425	0.000886	3.05256628	0.919	CTGACCCTGCC
MA0150.3	Nfe2l2	Foxa3	+	−881	−871	9.89E−05	4.00480371	0.685	gtgacacggcg
MA0150.3	Nfe2l2	Foxa3	+	−508	−498	0.000595	3.22548303	0.919	ctgactctgta
MA0150.3	Nfe2l2	Gale	+	−821	−811	0.000775	3.1106983	0.919	gtgagataaca
MA0150.3	Nfe2l2	Gkn3p	−	−158	−148	0.000624	3.20481541	0.919	ATGACTGAGTT
MA0150.3	Nfe2l2	Mgst2	−	−55	−45	0.000644	3.19111413	0.919	TTGAGTCAACA
MA0150.3	Nfe2l2	Mgst2	−	−914	−904	0.000906	3.0428718	0.919	GTGATGATGCA
MA0150.3	Nfe2l2	Muc6	−	−741	−731	0.00034	3.46852108	0.885	CTGAGTGAGCT
MA0150.3	Nfe2l2	Muc6	+	−233	−223	0.000375	3.42596873	0.885	ctgataaagca
MA0150.3	Nfe2l2	Muc6	+	−493	−483	0.000404	3.39361863	0.885	attacctagca
MA0150.3	Nfe2l2	Muc6	+	−398	−388	0.000588	3.23062267	0.919	gtgatctggca
MA0150.3	Nfe2l2	Nqo1	+	−436	−426	8.36E−06	5.07779372	0.347	gtgagtcggca
MA0150.3	Nfe2l2	Nqo1	+	−360	−350	0.000562	3.25026368	0.919	ctgagtgagga
MA0150.3	Nfe2l2	Nqo1	−	−448	−438	0.000829	3.08144547	0.919	GTGACTCTAGA
MA0150.3	Nfe2l2	Pglyrp1	+	−174	−164	0.000286	3.54363397	0.885	atgagagagcc
MA0150.3	Nfe2l2	Pglyrp1	−	−652	−642	0.000316	3.50031292	0.885	CTGAATCAGCC
MA0150.3	Nfe2l2	Pglyrp1	−	−388	−378	0.000496	3.30451832	0.919	ATGACTTTGAA
MA0150.3	Nfe2l2	Pglyrp1	+	−837	−827	0.000829	3.08144547	0.919	gtgacacatga
MA0150.3	Nfe2l2	Pglyrp1	−	−911	−901	0.000963	3.01637371	0.954	GTGACACTGGG
MA0150.3	Nfe2l2	Spdef	+	−692	−682	3.83E−05	4.41680123	0.398	gtgattcagct
MA0150.3	Nfe2l2	Tspan1	+	−640	−630	0.000395	3.4034029	0.885	gtgagccacca
MA0150.3	Nfe2l2	Tspan1	+	−449	−439	0.000395	3.4034029	0.885	gtgagccacca
MA0150.3	Nfe2l2	Tspan1	+	−213	−203	0.000404	3.39361863	0.885	ctgacactgct
MA0150.3	Nfe2l2	Tspan1	−	−567	−557	0.000713	3.14691047	0.919	GTGAGATGGCT
MA0150.3	Nfe2l2	Tspan1	−	−376	−366	0.000713	3.14691047	0.919	GTGAGATGGCT
MA0150.3	Nfe2l2	Tspan1	+	−747	−737	0.000856	3.06752624	0.919	gtgactgagtt
MA0150.3	Nfe2l2	Tspan1	+	−356	−346	0.000856	3.06752624	0.919	gtgactgagtt
MA0150.3	Nfe2l2	Wfdc2	−	−658	−648	2.99E−05	4.52432881	0.398	GTGACTATGCA
MA0150.3	Nfe2l2	Wfdc2	−	−765	−755	0.000282	3.54975089	0.885	ATGACTAAGGC

The table lists occurrences of the NRF2-binding motif (MA0150.1) within the promoters of genes in the human genome (GRCh38/hg38) and NRF2-binding motif (MA0150.2 and MA0150.3) within the promoters of genes in the mouse genome (GRCm38/mm10), filtered based on a *P*-value threshold of <0.001. Columns represent the following information: motif ID: identifier of the NRF2-binding motif. Alt ID: alternative ID for NRF2 (NFE2L2 or Nfe2l2). Sequence name: gene name associated with the identified motif. Strand: the DNA strand in which the motif was detected (+ for sense and − for antisense). Start/end: genomic coordinates indicating the position of the motif within the analyzed sequence. *P* value: statistical significance of the motif occurrence, representing the probability of a random sequence matching the motif with an equal or better score. *q* value: FDR-adjusted significance level for motif occurrences. Matched sequence: the specific nucleotide sequence matching the NRF2-binding motif at the given position.

Although further studies are needed to establish a direct functional relationship, these findings suggest that NRF2 loss enhances SPDEF and SPEM signatures in IPMN-like lesions, potentially promoting their development through a redox-independent regulatory mechanism. Given the emerging role of SPDEF in shaping an indolent, pyloric-like phenotype in IPMN, the NRF2–SPDEF axis may represent a key molecular distinction between PanIN and IPMN. Future investigations will be essential to determine whether NRF2 directly represses SPDEF activity and the influence of this regulatory network on lineage specification in pancreatic tumorigenesis.

## Discussion

Our study establishes a previously unrecognized role for NRF2 in suppressing IPMN formation distinct from its well-characterized tumor-promoting role in PanIN and PDA. Using a conditional knockout model, we observed that NRF2 deletion in KRAS-mutant pancreatic epithelium led to a marked increase in IPMN-like lesions despite suppressing PanIN formation. This dual role of NRF2 suggests that its function in pancreatic tumorigenesis is highly dependent on the context, potentially influencing lineage specification between PanIN and IPMN precursors.

IPMN is histologically distinct from PanIN, characterized by a mucinous epithelium that often resembles gastric or intestinal differentiation. Although NRF2 is known to promote tumor progression in pancreatic cancer (14, 15), it has also been implicated in tumor suppression in certain gastrointestinal malignancies, including chemically induced gastric (35) and colon (36) cancers. Our findings support a similar paradigm in pancreatic neoplasia, in which NRF2 deficiency influences pancreatic epithelial cell fate, favoring IPMN development through pathways associated with gastric differentiation. A recent study by DeBlasi and colleagues further highlighted the context-dependent role of NRF2, demonstrating that its requirement in lung tumorigenesis depends on the activation threshold of the pathway (37). Similarly, we observed a trend toward higher NRF2 expression in high-grade dysplastic IPMN compared with low-grade dysplastic IPMN, suggesting that the role of NRF2 in pancreatic precursor lesions may evolve with disease progression. Further studies are needed to delineate the contribution of NRF2 loss to early IPMN formation and the mechanisms driving its reactivation in malignant transformation.

The mechanism underlying NRF2 suppression of IPMN formation seems to be independent of its canonical role in redox homeostasis. Although NRF2 is widely recognized for its antioxidant functions (38), our findings showed that conventional approaches to alter intrapancreatic ROS levels did not affect IPMN incidence in KCN mice. Although future studies are required to examine whether the dose and duration of ROS perturbation might affect IPMN incidence, our current observation suggests that alternative NRF2-regulated pathways drive its tumor-suppressive function in IPMN. Supporting this, scRNA-seq analysis revealed that NRF2 loss led to significant enrichment of gene signatures associated with gastric SPEM- and SPDEF-driven transcriptional programs, implicating NRF2 in epithelial cell fate determination. SPDEF is a well-established transcription factor that regulates mucinous differentiation in the gastric and pulmonary epithelia (39, 40). Our motif analysis identified NRF2-binding AREs in key genes associated with SPEM- and SPDEF-driven pathways, including *Muc6*, *Spdef*, and *Cd44*, suggesting a direct role for NRF2 in modulating these transcriptional networks. Given that SPDEF has been linked to an indolent, pyloric-like phenotype in IPMN (12), our findings raise the possibility that NRF2 loss promotes IPMN formation by derepressing SPDEF activity, shifting pancreatic epithelial fate toward a mucinous differentiation program.

Beyond transcriptional regulation, NRF2 is a key modulator of metabolic reprogramming in cancer (38). IPMN, particularly those driven by *KRAS* and *GNAS* mutations, has been shown to rely on fatty acid oxidation (FAO) as a primary metabolic adaptation (41). As NRF2 has been reported to suppress FAO in other cancer contexts (42), its loss in pancreatic ductal cells may enable a FAO-driven metabolic shift that favors IPMN formation. Whether metabolic alterations contribute to NRF2-mediated lineage divergence between PanIN and IPMN remains an open question that warrants further investigation.

In summary, our study establishes NRF2 as a critical regulator of pancreatic precursor lesions, with distinct roles in PanIN and IPMN development. Unlike its pro-tumorigenic function in PanIN and PDA, NRF2 seems to suppress IPMN formation through redox-independent mechanisms, potentially by modulating transcriptional and metabolic programs associated with mucinous differentiation. Future studies will be needed to determine whether NRF2 directly represses SPDEF activity and to further explore the NRF2–SPDEF axis in pancreatic epithelial fate decisions. Understanding these mechanisms may provide new insights into the molecular divergence between PanIN and IPMN and identify potential vulnerabilities for early detection and targeted therapeutic strategies in pancreatic cancer.

## Supplementary Material

Figure S1 NRF2 and pNRF2 expression in IPMN

Figure S2 Histological spectrum of KC and KCN pancreata

Figure S3 Single-cell RNA sequencing analysis of KC and KCN pancreata
