# Supplementary material for: Dual Role of NRF2 in Pancreatic Precursor Lesions
Source: Cancer Res Commun. 2025 Jun 11;5(6):945–59. doi: 10.1158/2767-9764.CRC-25-0107 (PMC12158068; doi:10.1158/2767-9764.CRC-25-0107)
Supplement: Figure S1 — NRF2 and pNRF2 expression in IPMN [file crc-25-0107_figure_s1_suppsf1.pdf]

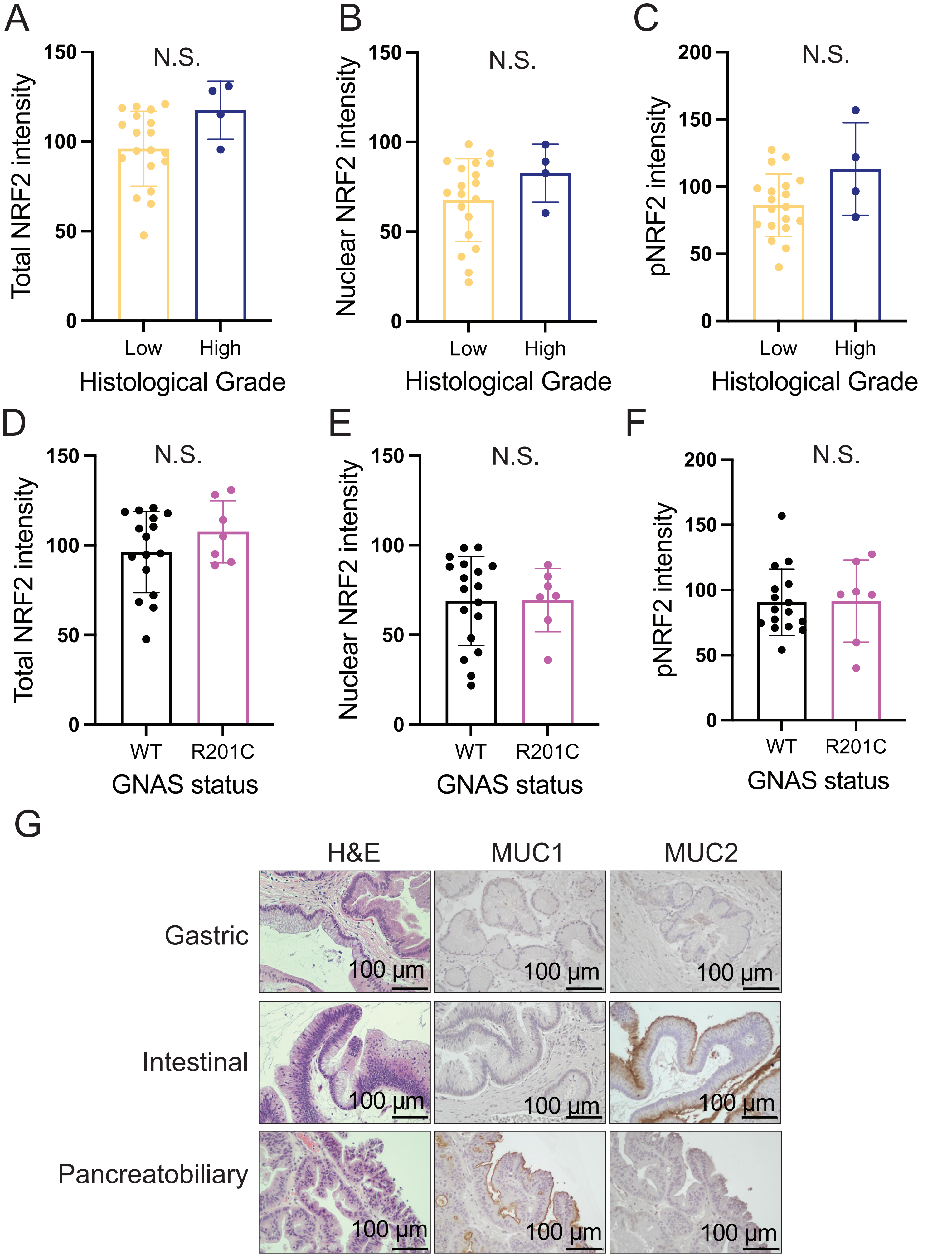

Supp Figure 1

Supplementary Figure. 1. NRF2 and pNRF2 expression in IPMN

A, B. Quantitative analysis of total (A) and nuclear (B) NRF2 intensity in LGD (n=19) and HGD (n=4) IPMN.

C. Quantitative analysis of pNRF2Ser40 intensity in LGD (n=19) and HGD (n=4) IPMN.

D, E. Quantitative analysis of total (D) and nuclear (E) NRF2 intensity in GNAS WT (n=7) and GNASR201C (n=16) specimens.

F. Quantitative analysis of pNRF2 intensity in Gnas wildtype (n=7) and GnasR201C (n=16) specimens.

G. Representative images of H&E, MUC1, and MUC2 immunohistochemical (IHC) staining of human IPMN specimens classified into different histology subtypes.

Error bars in this figure represent the means ± standard deviations. A Student's t-test was conducted. Unless stated otherwise, no significant differences were found between the two groups.
