# Supplementary material for: Dual Role of NRF2 in Pancreatic Precursor Lesions
Source: Cancer Res Commun. 2025 Jun 11;5(6):945–59. doi: 10.1158/2767-9764.CRC-25-0107 (PMC12158068; doi:10.1158/2767-9764.CRC-25-0107)
Supplement: Figure S2 — Histological spectrum of KC and KCN pancreata [file crc-25-0107_figure_s2_suppsf2.pdf]

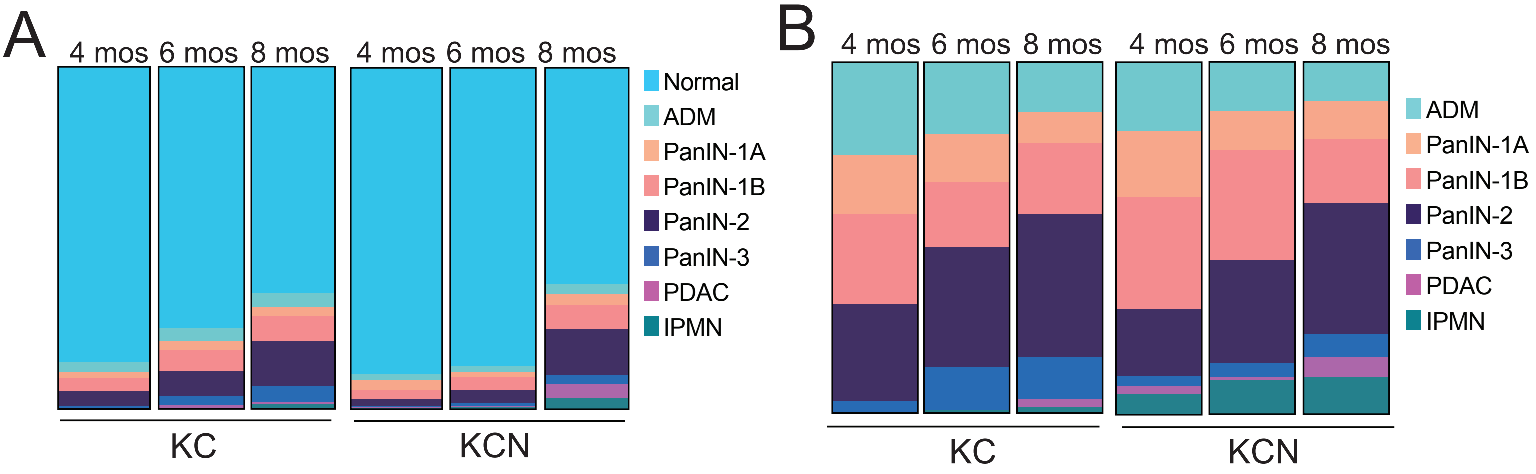

Supp Figure 2

Supplementary Figure. 2. Histological spectrum of KC and KCN pancreata

A, B. Percentage area of normal and neoplastic lesions calculated by dividing the area of normal tissue or neoplastic lesions by the total pancreatic area (A) or the total neoplastic areas (B). The cohorts included 25 KC and 15 KCN mice at 4 months, 23 KC and 11 KCN mice at 6 months, and 17 KC and 14 KCN mice at 8 months.
