# Supplementary material for: Dual Role of NRF2 in Pancreatic Precursor Lesions
Source: Cancer Res Commun. 2025 Jun 11;5(6):945–59. doi: 10.1158/2767-9764.CRC-25-0107 (PMC12158068; doi:10.1158/2767-9764.CRC-25-0107)
Supplement: Figure S3 — Single-cell RNA sequencing analysis of KC and KCN pancreata [file crc-25-0107_figure_s3_suppsf3.pdf]

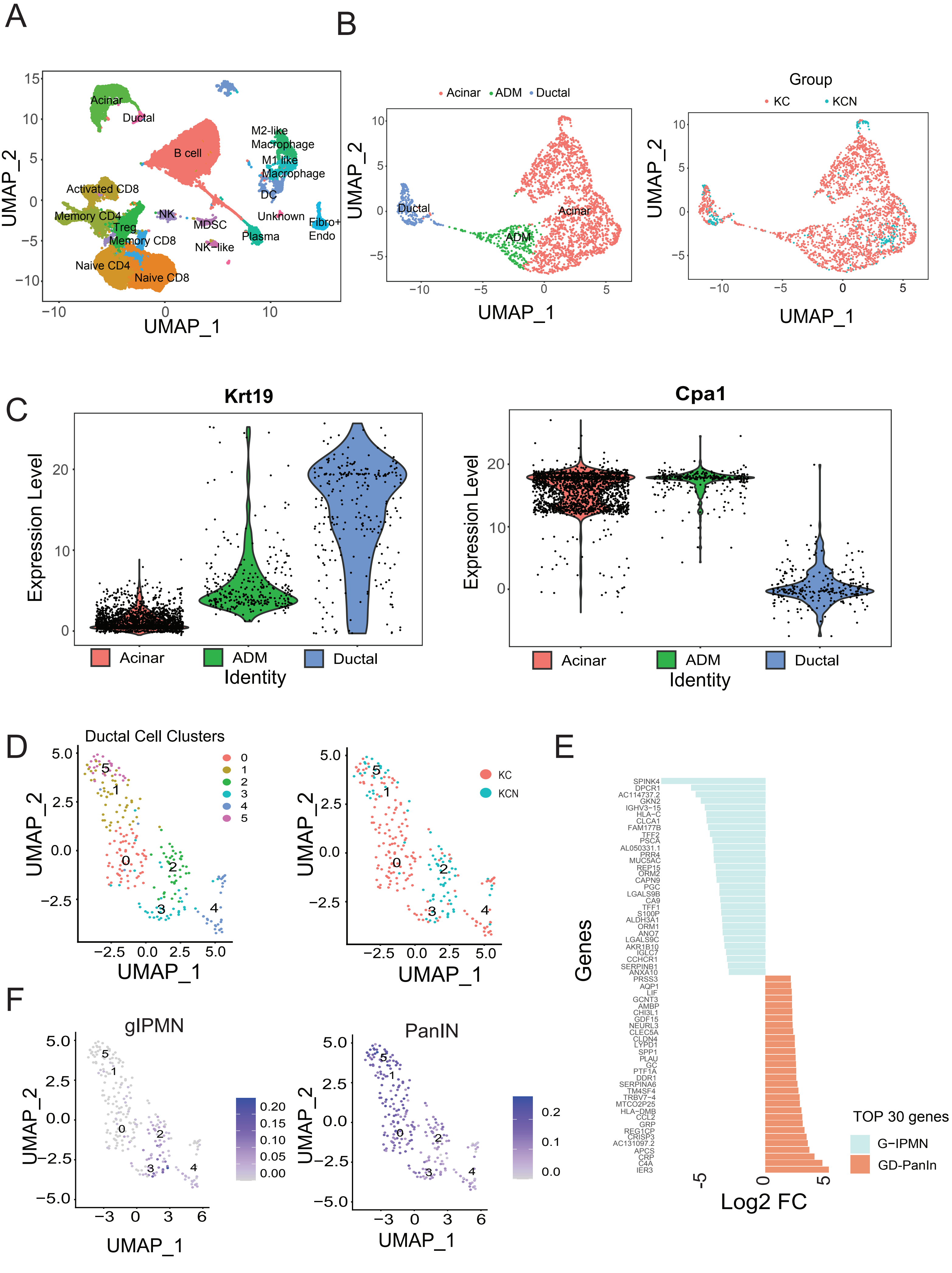

Supp Figure 3

Supplementary Figure. 3. Single-cell RNA sequencing analysis of KC and KCN pancreata

A. UMAP plot of 37,438 cells following quality control, normalization, and the removal of doublets and contaminants. Cell types were identified by analyzing the expression of established marker genes.

B. UMAP plot of 2,414 pancreatic epithelial cell clusters (left) and UMAP plot distinguishing the same cell population by KC and KCN group (right). The pancreatic epithelial cell population was generated by merging the ductal and acinar cell populations identified in Fig S3A.

C. Violin plot of Krt19 (Left) and Cpa1 (Right) expression across epithelial cell clusters

D. UMAP plot of Krt19+ Cpa1- ductal cell clusters (left) and UMAP plot distinguishing the same cell population by KC and KCNrf2flox group (right).

E. Top 30 differentially expressed genes (DEGs) from a public dataset (GSE210351) comparing human gastric IPMN and low-grade PanIN (LDG-PanIN).

F. UMAP plot of ductal cell clusters plotting the expression of gastric IPMN (gIPMN) and PanIN signatures.
